# Cancer- and Chemotherapy-Induced Changes in Cerebral Metabolism in Patients with Diffuse Large B-Cell Lymphoma: A Serial [^18^F]FDG PET Study

**DOI:** 10.3390/cancers17132222

**Published:** 2025-07-02

**Authors:** Insung Chung, Yeon-koo Kang, Jae Won Min, Seunggyun Ha, Joo Hyun O

**Affiliations:** Division of Nuclear Medicine, Department of Radiology, Seoul St. Mary’s Hospital, College of Medicine, The Catholic University of Korea, Seoul 06591, Republic of Korea; master990119@songeui.ac.kr (I.C.); yeonkoo11@gmail.com (Y.-k.K.);

**Keywords:** diffuse large B-cell lymphoma, chemotherapy, cancer-related cognitive impairment, FDG PET, brain metabolism, orbitofrontal cortex

## Abstract

**Background/Objectives**: Cancer-related cognitive impairment (CRCI) may result from both cancer and its treatment, complicating our understanding of the underlying mechanisms. This study aimed to distinguish cancer- and chemotherapy-induced cerebral metabolic changes in patients with diffuse large B-cell lymphoma (DLBCL) using [^18^F]FDG PET and to characterize their temporal dynamics during treatment, using images from healthy individuals as controls (HCs). **Methods**: We retrospectively analyzed [^18^F]FDG PET scans from DLBCL patients (n = 88) and age- and sex-matched HCs. Patient scans were obtained at three time points: baseline (Initial), after three cycles (Interim), and after six cycles of R-CHOP chemotherapy (end-of-treatment, EOT). Voxel-based analysis identified regions showing significant differences between groups or time points. Cancer-induced changes were defined as regions showing differences between patients and HCs at Initial but recovery by EOT. Chemotherapy-induced changes were defined as regions where the change from Initial to EOT in patients was consistent with that observed between HC and EOT. The identified regions were analyzed on a region of interest (ROI) basis for temporal trends across the time points. **Results**: The cancer-induced changes were diffusely decreased metabolic activity distributed across the cerebral cortex, with more prominent changes in the left inferior occipital and middle temporal gyri. They exhibited a quadratic temporal trend, with early rapid recovery and deceleration as treatment progressed. Chemotherapy-induced effects were localized to the bilateral orbitofrontal cortex (OFC) and exhibited a linear decline. **Conclusions**: Assessment of serial [^18^F]FDG PET from DLBCL patients and healthy individuals demonstrated (1) cancer-induced hypometabolism in diffuse cortical regions, which showed greater recovery earlier in the treatment course than later, and (2) R-CHOP chemotherapy-induced steadily decreasing metabolic activity in the bilateral OFC.

## Simple Summary

Cancer patients report cognitive problems during or after treatment, but whether these problems are caused by anxiety, cancer itself, or chemotherapy remains unclear. In this study, we used [^18^F]FDG PET images to track cerebral metabolic changes as surrogates of neural activity in patients with diffuse large B-cell lymphoma before, during, and after chemotherapy, using images of healthy individuals as controls. Cancer-induced changes appeared as reduced metabolism in widespread posterior cortical areas at diagnosis and showed early recovery during treatment. In contrast, chemotherapy-induced changes emerged in the bilateral orbitofrontal cortex during therapy and further declined at the end of therapy. These distinct patterns of changes in cerebral metabolic activity observed at different time points may help explain when and how cognitive problems could develop in patients with lymphoma.

## 1. Introduction

Varying degrees of cognitive impairment have been documented during the course of cancer treatment [1,2,3]. Thought to be caused by chemotherapy, the term “chemobrain” was previously used [4]. However, various therapeutic and symptomatic factors have been associated with cognitive impairment in cancer patients, and the newer term ‘cancer-related cognitive impairment (CRCI)’ more accurately reflects the broad range of potential causes such as radiation therapy, hormonal treatment, surgery, pain, and depression [2]. Moreover, underlying cancer itself was thought to contribute to CRCI in various types of cancer [5,6,7,8,9,10]. Inflammatory cytokines, brain-infiltrating immune cells, tumor-derived extracellular vesicles, injury to the integrity of the blood-brain barrier leading to direct toxicity to the central nervous system, oxidative stress in neuronal cells, and psychosocial and genetic factors have been suggested as the mechanisms of the CRCI [1,2,3,4,5]. However, it has not been possible to strictly distinguish the effects of cancer from the effects of chemotherapy on CRCI. Furthermore, we do not have a clear understanding of when the changes in cognitive function occur during the course of cancer treatment.

Previous neuroimaging studies using structural MRI, functional MRI, and PET demonstrated anatomical and functional changes associated with CRCI [11]. Notably, [^18^F]FDG PET images represent the sum of glucose transport and uptake at the molecular level and can thus serve as a proxy for neural activity in the brain.

In this study, by assessing [^18^F]FDG PET scans from patients with diffuse large B-Cell lymphoma (DLBCL) and healthy controls (HC), we aimed to distinguish changes in cerebral metabolism and thus cerebral neural activity caused by cancer from those induced by chemotherapy. Secondly, by comparing [^18^F]FDG PET scans obtained at three different time points during first-line therapy in the same patients, we aimed to track the temporal changes in cerebral metabolism over the course of treatment.

## 2. Materials and Methods

**Figure 1:**
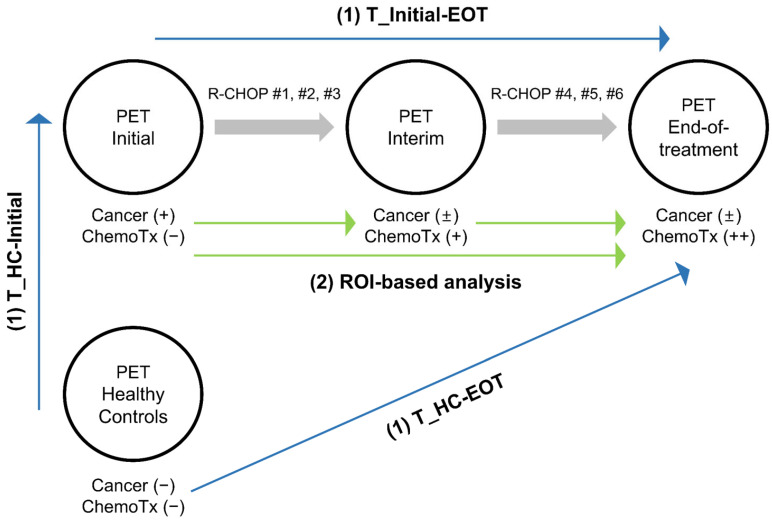
Paired sets of brain [^18^F]FDG PET images from DLBCL patients and a healthy control group were analyzed. (1) Voxel-based analysis (blue arrows): Voxel-wise *t*-tests were performed between each pair of PET datasets to generate *t*-statistic maps. Clusters identified from comparing the three *t*-statistic maps were subsequently used as ROIs for further analysis. (2) ROI-based analysis (green arrows): Mean SUVRs within these ROIs were statistically compared across the three PET datasets. Abbreviation: DLBCL, Diffuse large B-cell lymphoma; R-CHOP, rituximab, cyclophosphamide, hydroxydaunomycin, vincristine, prednisone regimen. Note: End-of-treatment, after 6-8 cycles for end of therapy assessment; Initial, baseline for staging; Interim, after 3-4 cycles of standard R-CHOP regimen for interim assessment; Cancer (+), presence of cancer; Cancer (±), mix of patients with active disease and those in remission; ChemoTx (−), no prior chemotherapy; ChemoTx (+) and ChemoTx (++), progressively increased exposure to chemotherapy.

To identify the clusters attributable to cancer and chemotherapy effects and to track temporal trends, we conducted a series of retrospective analyses summarized in Figure 1.

Three sets of [^18^F]FDG PET/CT scans were obtained from DLBCL patients at three time points: at baseline prior to therapy (PET_Initial), after 3 cycles of standard first-line therapy (PET_Interim), and at end-of-treatment after completing the first-line therapy (PET_EOT). An equal number of [^18^F]FDG PET/CT scans from age- and sex-matched cancer-free individuals were selected as the relatively healthy control group (PET_HC). From the systemic [^18^F]FDG PET/CT studies, PET images of the brain region were extracted for analyses.

(1) Voxel-based analysis: Three *t*-statistics maps were generated using three sets of PET scans. T_Initial-EOT demonstrates significant changes from PET_Initial to PET_EOT using a voxel-wise paired *t*-test. T_HC-Initial and T_HC-EOT demonstrate significant changes from PET_HC to PET_Initial and PET_EOT, respectively, using voxel-wise two-sample *t*-tests. By comparing these three *t*-statistics maps, clusters of changes in cerebral metabolism attributable mainly to cancer and others attributable mainly to chemotherapy were identified.
(2) Region of interest (ROI)-based analysis: The clusters identified by the voxel-based analysis were employed as ROIs, and we examined whether there were significant differences in cerebral metabolism within the ROIs at the three time points (PET_Initial, PET_Interim, and PET_EOT).

### 2.1. Participants

This study analyzed two cohorts: patients with diffuse large B-cell lymphoma (DLBCL) and age- and sex-matched cancer-free healthy controls.

For the DLBCL cohort, we retrospectively collected [^18^F]FDG PET/CT scans obtained at the Catholic Hematology Hospital of Seoul St. Mary’s Hospital between July 2014 and February 2021. The inclusion criteria were as follows: (1) patients pathologically diagnosed with DLBCL, (2) patients treated with R-CHOP (rituximab, cyclophosphamide, hydroxydaunomycin, vincristine, and prednisone) as the first-line chemotherapy, and (3) availability of PET scans at three time points—baseline for staging (Initial), after 3 cycles of the standard R-CHOP chemotherapy for interim assessment (Interim), and after 6 cycles for end-of-treatment assessment (EOT), all performed on the same PET/CT system. The exclusion criteria were as follows: (1) incomplete brain coverage in the scans, (2) presence of a brain lesion at any of the three time points, and (3) history of cerebrovascular, neurological, or psychiatric disorders or medication for these conditions. Ultimately, 264 PET scans from 88 patients were included in the analysis. Each patient underwent [^18^F]FDG PET/CT imaging at all three time points, yielding three PET datasets per patient, which were defined as PET_Initial, PET_Interim, and PET_EOT. In ten patients, however, the PET_Interim was obtained after four cycles of R-CHOP and the PET_EOT after up to eight cycles due to individualized variations.

For the HC cohort, we screened [^18^F]FDG PET/CT scans obtained at the Health Promotion Center of Seoul St. Mary’s Hospital between January 2009 and June 2024. The exclusion criteria were as follows: (1) incomplete brain coverage on the scan, (2) history of any malignant disease, (3) detection of a newly diagnosed malignancy on the scan, (4) visually detected abnormality in the brain region on the scan, and (5) history of cerebrovascular, neurological, or psychiatric disorders or medication history related to these conditions. From a total of 953 PET scans, 88 PET datasets (PET_HC) from 75 healthy subjects were selected to closely match the DLBCL patients in terms of age, and sex.

### 2.2. PET Image Acquisition & Reconstruction

After at least 6 h of fasting, [^18^F]FDG of 4.4 MBq/kg was intravenously administered. PET images were acquired 60 min post-injection, from above the skull vertex to mid-thighs or toes, from one of three dedicated PET/CT scanners: Discovery 710 (GE HealthCare, Chicago, IL, USA), Biograph40 (Siemens Healthineers, Erlangen, Germany), or Biograph 40 Truepoint (Siemens Healthineers, Erlangen, Germany). All three sets of the Initial, Interim, and EOT images were obtained using the Discovery 710 for the patients; the images of the HCs were obtained using all three scanners. Low-dose CT images were acquired immediately before PET acquisition for attenuation correction and anatomical localization. CT-based attenuation correction and scatter correction were applied. The matrix size was set to 192 × 192 for the Discovery 710 and 168 × 168 for Biograph 40 and Biograph 40 Truepoint. For subsequent analysis, axial PET slices covering the whole brain were extracted from each [^18^F]FDG PET/CT scan.

### 2.3. Image Preprocessing

Image preprocessing was performed using Statistical Parametric Mapping software 12 (SPM12; Wellcome Centre for Human Neuroimaging, London, UK), running under MATLAB R2024a (MathWorks Inc., Natick, MA, USA). Spatial normalization into the MNI 152 space was conducted using the PET template provided in SPM12, followed by smoothing using an 8 mm full-width at half maximum (FWHM) Gaussian filter. Voxel-wise intensity normalization was performed to generate standardized uptake value ratio (SUVR) maps, using the composite cerebellar area (labels 95–120) from the Automated Anatomical Labeling atlas 2 (AAL2) as the reference region [12]. Subsequently, cerebral areas (labels 1–94) were retained, and only gray matter regions were extracted by applying a threshold of 90% to the SPM gray matter probability map.

### 2.4. Voxel-Based Analysis

Voxel-wise *t*-tests were performed in SPM12 to compare three PET datasets, resulting in three statistical parametric maps of the *t*-statistics:

T_Initial-EOT: Changes from PET_Initial to PET_EOT, thought to represent metabolic changes occurring during the treatment course, induced by a combination of responding cancer and increasing chemotherapy cycles.

T_HC-Initial: Changes from PET_HC to PET_Initial, thought to represent the difference between relatively healthy brains and those of newly diagnosed lymphoma patients, possibly induced by cancer.

T_HC-EOT: Changes from PET_HC to PET_EOT, thought to represent the difference between relatively healthy brains and those of patients who completed first-line treatment, possibly induced by chemotherapy.

**Figure 2:**
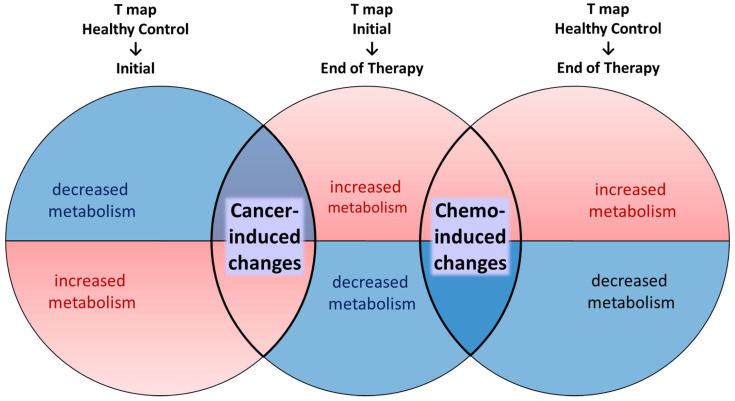
Schematic diagram illustrating the overlap between three *t*-statistics maps to identify cancer- and chemotherapy-induced metabolic changes. Red denotes increase in cerebral metabolism and blue denotes decrease in metabolism.

Paired *t*-tests were used for T_Initial-EOT, while two-sample *t*-tests were used for T_HC-Initial and T_HC-EOT. The height threshold was set at a false discovery rate (FDR) corrected *p* < 0.05, and an extent threshold of 200 voxels was applied to ensure reliable ROI size. To distinguish cancer- versus chemotherapy-induced metabolic changes, comparisons were made among the three *t*-statistics maps (Figure 2).

Clusters showing significant metabolic changes in opposite directions between T_HC-Initial (i.e., developed at time of cancer diagnosis) and T_Initial-EOT (i.e., reverted after treatment for cancer) were deemed to represent cancer-induced changes. Clusters with concordant metabolic changes in T_Initial-EOT (i.e., brought on during the course of treatment) and T_HC-EOT (i.e., not observed in healthy controls) were deemed to represent chemotherapy-induced changes. A minimum extent of 200 voxels was set for identification of the clusters. A detailed description of the comparison process is provided in the Supplementary Materials (Figure S1).

When the identified clusters were too large to precisely localize their anatomical locations, an additional analysis was performed using a height threshold of a family-wise error rate (FWE) corrected *p* < 0.05 instead of an FDR. The anatomical locations of the clusters were visualized and described using the xjView toolbox (https://www.alivelearn.net/xjview, accessed on 7 August 2024).

### 2.5. ROI-Based Analysis

A repeated measures analysis of variance (ANOVA) was used to assess differences in mean SUVRs in ROIs—clusters identified by the voxel-based analysis—across the three time points. Prior to performing the repeated measures ANOVA, the assumption of sphericity was tested by Mauchly’s test. When sphericity was violated (*p* < 0.05), the degrees of freedom were adjusted using Greenhouse–Geisser correction. Polynomial contrasts were used to investigate trends across the three time points: Initial vs. Interim vs. EOT. Multiple comparisons were adjusted using the FDR method for multiple ANOVA tests and polynomial contrasts. The Bonferroni method was used when adjusting for post-hoc pairwise comparisons among time points. Statistical significance was defined as a two-tailed *p* < 0.05.

### 2.6. Statistical Analysis

Descriptive statistics, two-sample *t*-tests, paired *t*-tests, and sign tests were performed for continuous variables. Statistical significance was defined as a two-tailed *p* < 0.05. All statistical analyses were performed using the Statistics and Machine Learning Toolbox in MATLAB R2024a (MathWorks Inc., Natick, MA, USA).

### 2.7. Use of Artificial Intelligence

During the preparation of this work, the authors used ChatGPT (4 March 2025 version, OpenAI) for assistance with generating analysis scripts and improving the clarity of English writing. After using this tool, the authors reviewed and revised the content as needed, and we take full responsibility for the content of the published article.

## 3. Results

### 3.1. Demographics and Clinical Characteristics

The mean age of DLBCL patients (58.8 ± 15.5 years) at baseline (i.e., PET_Initial) did not significantly differ from that of the HCs (58.8 ± 9.9 years), and the sex distribution was identical between the two groups (male:female, 58:30). The mean time interval from PET_Initial to PET_Interim was 73.5 ± 11.7 days (range: 56–126), and did not exhibit significant differences in interval from PET_Interim to PET_EOT (76.4 ± 19.4 days; range: 55–185). The Ann-Arbor stage at the time of diagnosis was stage I–II in 49 patients and III–IV in 39 patients. Regarding treatment response, at PET_EOT 81 patients (92%) showed complete response (CR) (Table 1).

### 3.2. Cancer-Induced Changes in Cerebral Metabolism

**Figure 3:**
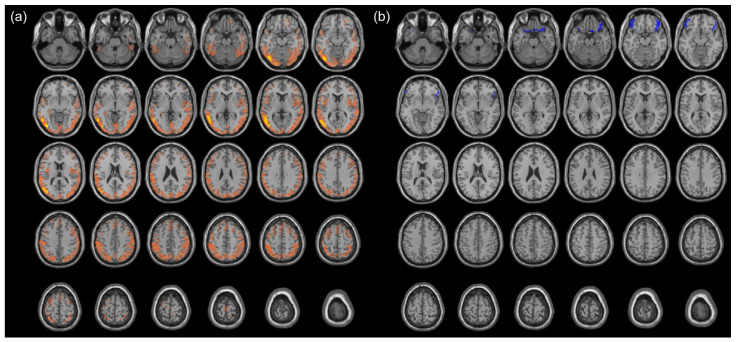
Clusters of cancer-induced and chemotherapy-induced changes in cerebral metabolism in DLBCL patients. Clusters that show cancer-induced decreases in cerebral metabolism identified by comparison of T_Initial-EOT and T_HC-Initial, are shown. (**a**), Clusters significant at FDR-corrected *p* < 0.05 are depicted in orange; Clusters significant at FWE-corrected *p* < 0.05 are depicted in yellow. (**b**) Clusters that show chemotherapy-induced decreases in cerebral metabolism following R-CHOP chemotherapy, identified by comparison of T_Initial-EOT and T_HC-EOT, are shown in blue (FDR-corrected *p* < 0.05). Abbreviations: EOT, end of treatment; FDR, false discovery rate; FWE, family-wise error; HC, healthy control.

Changes in cerebral metabolism were first assessed using voxel-wise comparisons between the DLBCL patients at baseline and the HCs (i.e., PET_Initial vs. PET_HC), applying an FDR-corrected threshold. At PET_Initial, patients with DLBCL exhibited significantly decreased cerebral metabolism in widespread areas across the temporal, parietal, and occipital cortices compared to the HCs. Among these areas, a substantial portion showed significant recovery at PET_EOT, representing cancer-induced changes (orange areas in Figure 3a). To more precisely identify the regions most affected by the presence of malignancy, an additional voxel-wise analysis was performed using an FWE-corrected threshold. The cancer-induced metabolic decreases were most pronounced in the left inferior occipital gyrus and left middle temporal gyrus (coordinates in Table S1). No cluster was observed in which an increased cerebral metabolism could be attributed to cancer.

The CR group (n = 81) showed greater recovery from cancer-induced hypometabolism compared to the non-CR group (n = 7), but the difference was not statistically significant (*p* = 0.262 and *p* = 0.160 from the FDR-corrected cluster and the FWE-corrected cluster, respectively).

### 3.3. Chemotherapy-Induced Change in Cerebral Metabolism

Changes in cerebral metabolism were first assessed using voxel-wise comparisons between DLBCL patients at EOT and the HCs (i.e., PET_EOT vs. PET_HC), applying an FDR-corrected threshold. At PET_EOT, patients with DLBCL exhibited significantly decreased cerebral metabolism in the bilateral orbitofrontal cortex (OFC) and increased metabolism in the left cuneus. Among these areas, concordant findings were seen from PET_Initial to PET_EOT in the bilateral OFC (significantly decreased metabolism), representing chemotherapy-induced effects (blue areas in Figure 3b). The most pronounced metabolic changes were observed in the right gyrus rectus and the left lateral orbital gyrus (coordinates in Table S2). No cluster was observed in which an increased cerebral metabolism could be attributable to chemotherapy.

The chemotherapy-induced hypometabolism was less severe in the CR group compared to the non-CR group, but the difference was not statistically significant (*p* = 0.154 from the FDR-corrected cluster).

### 3.4. Temporal Patterns of Cancer- and Chemotherapy-Induced Metabolic Changes

The clusters identified in the voxel-based analysis were used as ROIs, and their mean SUVRs were compared across the three time points. For ROIs classified as cancer-induced, both the FDR- and FWE-corrected clusters were analyzed.

**Figure 4:**
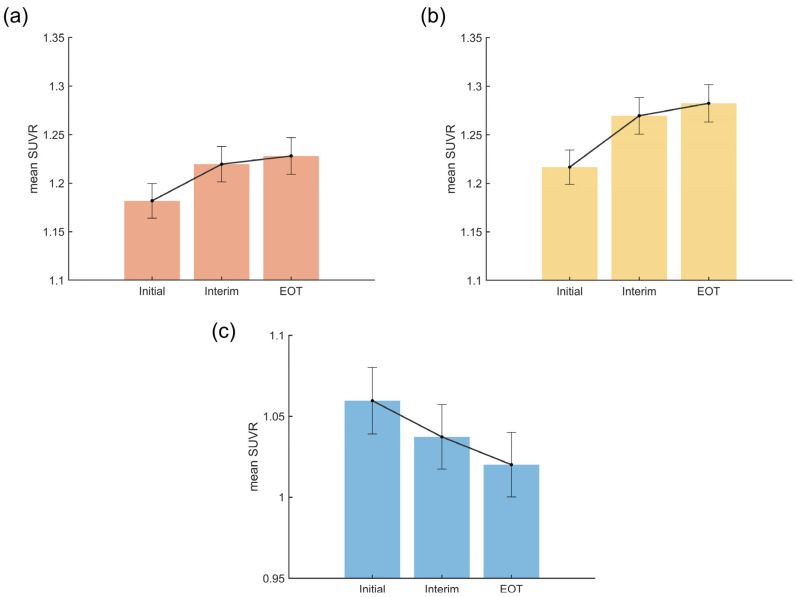
Cancer-induced and chemotherapy-induced changes in cerebral metabolism represented as mean SUVR in DLBCL patients across three time points (Initial vs. Interim vs. EOT). (**a**) Cancer-induced (FDR-corrected clusters); (**b**) Cancer-induced (FWE-corrected clusters); (**c**) Chemotherapy-induced (FDR-corrected clusters). Abbreviations: DLBCL, diffuse large B-cell lymphoma; EOT, end of treatment; FDR, false discovery rate; FWE, family-wise error; SUVR, standardized uptake value ratio.

In the FDR-corrected cancer-induced ROIs, repeated measures ANOVA revealed a significant main effect of time (*F* = 28.58, *p* < 0.001, η^2^ = 0.25), with post-hoc pairwise comparisons showing significant increases from PET_Initial to both PET_Interim and PET_EOT, but no significant differences between the latter two time points (Table 2). Mean SUVRs increased from PET_Initial (1.18 ± 0.08) to PET_Interim (1.22 ± 0.09) to PET_EOT (1.23 ± 0.09) (Figure 4a). Both the linear (*t* = 6.31, *p* < 0.001) and quadratic (*t* = −3.03, *p* = 0.005) contrasts were statistically significant (Table 3).

In the smaller FWE-corrected cancer-induced ROIs, results similar to the broader FDR-corrected clusters were observed: a main effect of time (Table 2), increasing mean SUVRs (Figure 4b), and a quadratic trend of metabolic recovery (Table 3).

For ROIs classified as chemotherapy-induced, a significant time effect (*F* = 27.75, *p* < 0.001, η^2^ = 0.24) and progressive decline across time points were observed (Table 2). The mean SUVRs progressively decreased from PET_Initial (1.06 ± 0.10) to PET_Interim (1.04 ± 0.09) to PET_EOT (1.02 ± 0.09) (Figure 4c), and only the linear contrast was statistically significant (*t* = −6.67, *p* < 0.001), indicating a linear decline over time (Table 3).

## 4. Discussion

In this study, three sets of [^18^F]FDG PET scans (Initial, Interim, and EOT) from patients with DLBCL and [^18^F]FDG PET scans from age- and sex- matched HCs were analyzed. Our results can be summarized as follows: (1) cancer-induced changes were seen as diffusely decreased metabolic activity across the cerebral cortex, especially in the left inferior occipital and middle temporal gyri. There was a quadratic trend in the recovery pattern: a sharp increase from Initial to Interim, followed by a modest rise to EOT. (2) Chemotherapy-induced changes were confined to the bilateral orbitofrontal cortex, showing a linear decline from Initial to Interim to EOT.

CRCI can occur at various points during the process of cancer diagnosis and treatment. In a review of previous studies, CRCI was observed in 20–40% of patients before treatment, 65–75% during treatment, and 30–60% long-term after treatment [2].

Our results of cancer-induced metabolic changes may explain the CRCI observed at the time of diagnosis even before treatment begins. Previous studies have also demonstrated that the reduced brain metabolism seen in malignant lymphoma patients without brain lesions recovers in good responders after treatment [13,14]. One could argue that the competitive utilization of FDG in the high volume of tumor lesions rather than a true decrease in cerebral neural activity can appear as lower FDG uptake in the brain. The observation that the liver also exhibited decreased uptake at baseline could support this point [14]. However, unlike previous studies that analyzed the maximum or average SUV within brain ROIs, we estimated cerebral glucose metabolism using SUVR values normalized to the whole cerebellum, and this method should provide stable, internally corrected values of cerebral activity over varying clinical conditions. It should be pointed out that even if competition for FDG with a tumor was responsible for the low cerebral activity, the effect was not equal throughout the brain, since the left inferior occipital gyrus and left middle temporal gyrus were more ready to forgo FDG than other cortical areas in our study. Moreover, multiple studies with varying tumor volumes and tumor types, including myeloid disorders with known low FDG-avidity on PET, have reported cognitive impairment in cancer patients before any treatment is administered (including surgery, radiation therapy, and hormonal treatment) [5,6,7,8,9], suggesting that cancer itself could contribute to neurocognitive alterations either directly or indirectly. In addition, recent research suggests that an elevation in blood lactate levels in malignant lymphoma may lead to a decrease in brain glucose utilization [15].

Our results of chemotherapy-induced changes may explain the worsening of CRCI observed during treatment. Several studies using brain [^18^F]FDG PET and functional MRI in cancer patients have reported decreases in OFC metabolism and activity following chemotherapy [16,17,18,19], and post-chemotherapy cognitive decline was associated with reductions in OFC metabolism and activity in breast cancer [18,19,20]. The OFC is known to integrate sensory, limbic, and prefrontal cognitive information to support complex tasks [21]. It plays a critical role in decision-making [22] and in representing reward value and emotion, which may contribute to affective symptoms such as depression [23]. CRCI primarily affects attention, processing speed, executive function, and memory [1,2]. Additionally, cancer patients often experience depression and anxiety during diagnosis and treatment, which are linked to psychomotor slowing, inefficient learning, and executive dysfunction, which are common features of CRCI [2]. Moreover, some research shows that affective distress correlates more strongly with patients’ subjective cognitive complaints than with objective neuropsychological test results [24]. In summary, metabolic reduction in the OFC may be linked to CRCI in two ways. First, it may impair the OFC’s decision-making ability, directly contributing to cognitive impairment. Second, it may reflect the OFC’s role in emotional regulation, with cognitive impairment often accompanied by the affective symptoms seen in cancer patients.

The distinct quadratic recovery of cancer-induced changes and linear decline in chemotherapy-induced metabolic changes captures the underlying biological processes—rapid tumor response versus cumulative chemotoxicity. Specifically, recovery from cancer-induced hypometabolism occurred predominantly from Initial to Interim. Considering that there were no significant differences in the time intervals between the Initial-to-Interim and Interim-to-EOT periods, this finding may reflect the pattern of decline in total tumor burden [13,25]. Interim response evaluation in DLBCL treatment is a predictor of outcome and prognosis, with good responders often showing marked reductions in tumor burden early in the treatment course compared to later stages [26,27,28,29]. In contrast, chemotherapy-induced changes occurred in a linear fashion across the three time points, which likely reflects the consistent number of treatment cycles administered between these time points. The chemotherapy-induced linear reduction in cerebral metabolism likely mirrors the cumulative exposure to chemotherapeutic agents, with metabolic impairment scaling directly with the number of cycles administered.

### 4.1. Limitations

Our study is a retrospective investigation of cancer patients and preventive health check-up participants from a single center, and it has several limitations: (1) Cognitive function was not evaluated, thereby limiting the clinical interpretation of our findings. (2) Our cancer cohort was limited to newly diagnosed DLBCL patients, which may not reflect CRCI that occurs across various cancer types and treatment protocols. (3) The population participating in our center’s health check-ups may not represent the general population in terms of socioeconomic status and lifestyle habits. Our analysis methodology has several limitations in data processing and interpretation. (1) The brain PET images we analyzed were extracted from [^18^F]FDG PET/CT images of the torso or whole body obtained for oncologic evaluation, and did not meet the scan time and reconstruction factors recommended for dedicated brain images. Due to the lower number of counts and resolution of the images used for assessment in this study, the quality of data cannot be guaranteed. We are currently prospectively collecting dedicated brain PET images for comparison with the data used in this study. (2) The entire analysis is based on the assumption that changes in brain metabolism during chemotherapy result from a combination of the resolution of cancer-induced effects and the direct effects of chemotherapy, without fully accounting for regions influenced by either both factors or their potential interactions. This assumption may oversimplify the underlying neurobiological processes. (3) The process of deriving clusters by overlapping three *t*-statistics maps is highly dependent on the chosen thresholds and overlapping rules, which are somewhat arbitrary. (4) Data heterogeneity due to the presence of uncontrolled confounding variables (e.g., comorbidities and medication history) could not be avoided, and led us to use normalized SUVR as a compensation method.

### 4.2. Directions for Future Research

Ideally, future research could integrate neurocognitive and psychological assessments to investigate how alterations observed in functional brain imaging relate to or diverge from clinical manifestations. A recent study showed that functional brain images need not be related to clinical manifestations [30]. In another study, the fMRI findings showed a stronger correlation with subjective cognitive concerns than with objective cognitive tests [31]. Various cancer types and treatments should be investigated with multimodal imaging techniques and biomarkers to clarify the complex effects of cancer and its treatments on CRCI. Given that [^18^F]FDG PET/CT is already widely used for staging and response evaluation in many types of cancer, its potential as a marker for CRCI risk and a tool for targeted cognitive interventions should be explored in prospective studies. The total tumor volume and metabolic activity in the tumors could also be measured and included to gauge the effect of tumor burden on CRCI. The use of [^18^F]FDG PET/CT with cognitive testing may facilitate the development of neuroprotective strategies for cancer patients.

## 5. Conclusions

Our findings revealed cancer-induced hypometabolism in diffuse cortical regions and chemotherapy-induced hypometabolism localized to the bilateral OFC in patients with DLBCL treated with R-CHOP. While cancer-induced effects showed a quadratic pattern of recovery, chemotherapy-induced changes followed a linear decline across treatment. These distinct spatial and temporal patterns may help explain the neural mechanisms underlying cancer-related cognitive impairment, but additional prospective studies across various cancer and treatment types are needed.

## Figures and Tables

**Table 1:** Demographics and Clinical characteristics.

	HC (n = 88)	DLBCL (n = 88)	Test Statistics	*p* Value
Demographics				
Age, years	58.8 ± 9.9	58.8 ± 15.5	0.02 ^†^	0.982
Sex, Male, Female	58, 30	58, 30	-	-
Ann-Arbor stage, I–II, III–IV		49, 39		
Time Intervals				
Initial to Interim (days)	-	73.5 ± 11.7	−1.26 ^‡^	0.210
Interim to EOT (days)	-	76.4 ± 19.4		
Treatment Cycles				
Initial to Interim (cycles)	-	3.1 ± 0.2	5 ^§^	0.453
Interim to EOT (cycles)	-	3.0 ± 0.4		
Response at EOT, CR, non-CR	-	81, 7		

Data is shown as mean ± standard deviation unless remarked otherwise. Note: ^†^, Two-sample *t*-test (HC vs. DLBCL); ^‡^, Paired *t*-test (Initial-Interim vs. Interim-EOT); ^§^, Sign test (Initial-Interim vs. Interim-EOT). Abbreviations; DLBCL, diffuse large B-cell lymphoma; EOT, after 6 cycles for end-of-therapy assessment; HC; healthy control; Initial, baseline for staging; Interim, after 3 cycles of standard R-CHOP regimen for interim assessment; R-CHOP, rituximab, cyclophosphamide, hydroxydaunomycin, vincristine, prednisone regimen.

**Table 2:** Regional standardized uptake value ratio (SUVR) differences across three time points.

Regional SUVR	Initial ^a^ (n = 88)	Interim ^b^ (n = 88)	EOT ^c^ (n = 88)	*F*	*p*-Value_adj_	η^2^	Pairwise Comparison
Cancer-induced (FDR)	1.18 ± 0.08	1.22 ± 0.09	1.23 ± 0.09	28.58	2.78 × 10^−11^	0.25	^a^ < ^b^ ***, ^a^ < ^c^ ***
Cancer-induced (FWE)	1.22 ± 0.08	1.27 ± 0.09	1.28 ± 0.09	36.35	1.93 × 10^−13^	0.30	^a^ < ^b^ ***, ^a^ < ^c^ ***
Chemotherapy-induced (FDR)	1.06 ± 0.10	1.04 ± 0.09	1.02 ± 0.09	27.75	3.47 × 10^−11^	0.24	^a^ > ^b^ ***, ^a^ > ^c^ ***, ^b^ > ^c^ **

Data are shown as mean ± standard error. Repeated measures analysis of variance was performed. FDR correction was applied to *p*-values across regions. Test statistics of *F* are labeled. Eta squared (η^2^) estimates are shown for effect sizes. Bonferroni correction was applied to post-hoc pairwise comparisons. ^a^, Initial, baseline for staging; ^b^, Interim, after 3 cycles of standard chemotherapy; ^c^, EOT, after 6 cycles for end-of-treatment assessment; ** *p* < 0.01; *** *p* < 0.001. _adj_, adjusted; FDR, false discovery rate; FWE, family-wise error.

**Table 3:** Regional standardized uptake value ratio (SUVR) trend across time points (Initial vs. Interim vs. EOT).

Regional SUVR	Contrast	Estimate	SE	*t*	*p* Value_adj_
Cancer-induced (FDR)	Linear ***	0.046	0.007	6.31	1.70 × 10^−8^
	Quadratic **	−0.029	0.010	−3.03	0.005
Cancer-induced (FWE)	Linear ***	0.066	0.009	7.28	4.21 × 10^−10^
	Quadratic **	−0.040	0.012	−3.20	0.006
Chemotherapy-induced (FDR)	Linear ***	−0.040	0.006	−6.67	2.88 × 10^−9^
	Quadratic	0.005	0.008	0.66	0.514

Polynomial contrasts were performed. FDR correction was applied to *p*-values across regions. ** *p* < 0.01; *** *p* < 0.001. _adj_, adjusted; FDR, false discovery rate; FWE, family-wise error; SE, standard error.

## Data Availability

The data that support the findings of this study and codes for statistical analyses are available from the corresponding author upon reasonable request. Permission from our institution’s Data Review Board is requisite prior to sharing (contact to ojoohyun@songeui.ac.kr).

## Supplementary Materials

The following supporting information can be downloaded at: https://www.mdpi.com/article/10.3390/cancers17132222/s1, Table S1: Peaks of the clusters of cancer-induced changes in cerebral metabolism in DLBCL patients; Table S2: Peaks of the clusters of chemotherapy-induced changes in cerebral metabolism in DLBCL patients; Figure S1: Conceptual diagram illustrating cancer- and chemotherapy-induced changes in cerebral metabolism across the three *t*-statistic maps.

## References

[1] Lange M., Joly F., Vardy J., Ahles T., Dubois M., Tron L., Winocur G., De Ruiter M.B., Castel H. (2019). Cancer-related cognitive impairment: An update on state of the art, detection, and management strategies in cancer survivors. Ann. Oncol..

[2] Vannorsdall T.D. (2017). Cognitive Changes Related to Cancer Therapy. Med. Clin. N. Am..

[3] Haywood D., Dauer E., Baughman F.D., Lawrence B.J., Rossell S.L., Hart N.H., O’Connor M. (2023). “Is My Brain Ever Going to Work Fully Again?”: Challenges and Needs of Cancer Survivors with Persistent Cancer-Related Cognitive Impairment. Cancers.

[4] Hurria A., George S., Ahles T. (2007). Renaming “Chemobrain”. Cancer Investig..

[5] Meyers C.A., Byrne K.S., Komaki R. (1995). Cognitive deficits in patients with small cell lung cancer before and after chemotherapy. Lung Cancer.

[6] Meyers C.A., Albitar M., Estey E. (2005). Cognitive impairment, fatigue, and cytokine levels in patients with acute myelogenous leukemia or myelodysplastic syndrome. Cancer.

[7] Vardy J.L., Dhillon H.M., Pond G.R., Rourke S.B., Bekele T., Renton C., Dodd A., Zhang H., Beale P., Clarke S. (2015). Cognitive Function in Patients with Colorectal Cancer Who Do and Do Not Receive Chemotherapy: A Prospective, Longitudinal, Controlled Study. J. Clin. Oncol..

[8] Baekelandt B.M., Hjermstad M.J., Nordby T., Fagerland M.W., Kure E.H., Heiberg T., Buanes T., Labori K.J. (2016). Preoperative cognitive function predicts survival in patients with resectable pancreatic ductal adenocarcinoma. HPB.

[9] Hshieh T.T., Jung W.F., Grande L.J., Chen J., Stone R.M., Soiffer R.J., Driver J.A., Abel G.A. (2018). Prevalence of Cognitive Impairment and Association with Survival Among Older Patients with Hematologic Cancers. JAMA Oncol..

[10] Olson B., Marks D.L. (2019). Pretreatment Cancer-Related Cognitive Impairment-Mechanisms and Outlook. Cancers.

[11] Simo M., Rifa-Ros X., Rodriguez-Fornells A., Bruna J. (2013). Chemobrain: A systematic review of structural and functional neuroimaging studies. Neurosci. Biobehav. Rev..

[12] Rolls E.T., Joliot M., Tzourio-Mazoyer N. (2015). Implementation of a new parcellation of the orbitofrontal cortex in the automated anatomical labeling atlas. Neuroimage.

[13] Nonokuma M., Kuwabara Y., Takano K., Tamura K., Ishitsuka K., Yoshimitsu K. (2014). Evaluation of regional cerebral glucose metabolism in patients with malignant lymphoma of the body using statistical image analysis. Ann. Nucl. Med..

[14] Otomi Y., Otsuka H., Shono N., Onishi H., Mitsuhashi R., Matsuzaki S., Takaoka Y., Enomoto H., Sakamoto Y., Sasahara M. (2021). A reduced physiological (18)F-fluorodeoxyglucose uptake in the brain and liver caused by malignant lymphoma being deprived of the tracer. J. Med. Investig..

[15] Yi H.K., Yoo J., Kim S.J., Choi J.Y., Lee K.H. (2022). Lymphoma total lesion glycolysis leads to hyperlactatemia and reduction of brain glucose utilization. Sci. Rep..

[16] Hu Y., Zhang Q., Cui C., Zhang Y. (2022). Altered Regional Brain Glucose Metabolism in Diffuse Large B-Cell Lymphoma Patients Treated with Cyclophosphamide, Epirubicin, Vincristine, and Prednisone: An Fluorodeoxyglucose Positron Emission Tomography Study of 205 Cases. Front. Neurosci..

[17] Chiaravalloti A., Pagani M., Cantonetti M., Pietro B.D., Tavolozza M., Travascio L., Biagio D.D., Danieli R., Schillaci O. (2015). Brain metabolic changes in Hodgkin disease patients following diagnosis and during the disease course: An (18)F-FDG PET/CT study. Oncol. Lett..

[18] Ponto L.L., Menda Y., Magnotta V.A., Yamada T.H., Denburg N.L., Schultz S.K. (2015). Frontal hypometabolism in elderly breast cancer survivors determined by [(18)F]fluorodeoxyglucose (FDG) positron emission tomography (PET): A pilot study. Int. J. Geriatr. Psychiatry.

[19] López Zunini R.A., Scherling C., Wallis N., Collins B., MacKenzie J., Bielajew C., Smith A.M. (2013). Differences in verbal memory retrieval in breast cancer chemotherapy patients compared to healthy controls: A prospective fMRI study. Brain Imaging Behav..

[20] Menning S., de Ruiter M.B., Veltman D.J., Boogerd W., Oldenburg H.S., Reneman L., Schagen S.B. (2017). Changes in brain activation in breast cancer patients depend on cognitive domain and treatment type. PLoS ONE.

[21] Haber S.N., Behrens T.E. (2014). The neural network underlying incentive-based learning: Implications for interpreting circuit disruptions in psychiatric disorders. Neuron.

[22] Wallis J.D. (2007). Orbitofrontal cortex and its contribution to decision-making. Annu. Rev. Neurosci..

[23] Rolls E.T., Cheng W., Feng J. (2020). The orbitofrontal cortex: Reward, emotion and depression. Brain Commun..

[24] Pullens M.J., De Vries J., Roukema J.A. (2010). Subjective cognitive dysfunction in breast cancer patients: A systematic review. Psychooncology.

[25] Hanaoka K., Hosono M., Shimono T., Usami K., Komeya Y., Tsuchiya N., Yamazoe Y., Ishii K., Tatsumi Y., Sumita M. (2010). Decreased brain FDG uptake in patients with extensive non-Hodgkin’s lymphoma lesions. Ann. Nucl. Med..

[26] Park H.L., Han E.J., O J.H., Choi B.O., Park G., Jung S.E., Yahng S.A., Eom K.S., Cho S.G., on behalf of Catholic University Lymphoma Group (2020). Early Interim Chemotherapy Response Evaluation by F-18 FDG PET/CT in Diffuse Large B Cell Lymphoma. Diagnostics.

[27] Zhu L., Meng Y., Guo L., Zhao H., Shi Y., Li S., Wang A., Zhang X., Shi J., Zhu J. (2021). Predictive value of baseline (18)F-FDG PET/CT and interim treatment response for the prognosis of patients with diffuse large B-cell lymphoma receiving R-CHOP chemotherapy. Oncol. Lett..

[28] Lin C., Itti E., Haioun C., Petegnief Y., Luciani A., Dupuis J., Paone G., Talbot J.N., Rahmouni A., Meignan M. (2007). Early 18F-FDG PET for prediction of prognosis in patients with diffuse large B-cell lymphoma: SUV-based assessment versus visual analysis. J. Nucl. Med..

[29] Malek E., Sendilnathan A., Yellu M., Petersen A., Fernandez-Ulloa M., Driscoll J.J. (2015). Metabolic tumor volume on interim PET is a better predictor of outcome in diffuse large B-cell lymphoma than semiquantitative methods. Blood Cancer J..

[30] Apple A.C., Schroeder M.P., Ryals A.J., Wagner L.I., Cella D., Shih P.A., Reilly J., Penedo F.J., Voss J.L., Wang L. (2018). Hippocampal functional connectivity is related to self-reported cognitive concerns in breast cancer patients undergoing adjuvant therapy. NeuroImage Clin..

[31] Kardan O., Reuter-Lorenz P.A., Peltier S., Churchill N.W., Misic B., Askren M.K., Jung M.S., Cimprich B., Berman M.G. (2019). Brain connectivity tracks effects of chemotherapy separately from behavioral measures. NeuroImage Clin..

