# Supplementary material for: Cancer- and Chemotherapy-Induced Changes in Cerebral Metabolism in Patients with Diffuse Large B-Cell Lymphoma: A Serial [18F]FDG PET Study
Source: Cancers (Basel). 2025 Jul 2;17(13):2222. doi: 10.3390/cancers17132222 (PMC12248696; doi:10.3390/cancers17132222)
Supplement: Supplementary file 1 [file cancers-17-02222-s001.zip › cancers-3700421-supplementary.pdf]

Supplementary Materials

1. Supplementary Tables

Table S1. Peaks of the clusters of cancer-induced changes in cerebral metabolism in DLBCL patients.

Brain Region (AAL2)	MNI coordinates			*t*	***p*-value$_{adj}$**	Cluster size
	x	y	z			
Inferior occipital gyrus (L)	-38	-78	-6	8.94	< 0.001	293
Middle temporal gyrus (L)	-52	-70	10	7.34	< 0.001	601

The x, y, z = coordinates according to the MNI stereotactic space for clusters showing the effects attributable mainly to cancer, in the AAL2 atlas (cerebellum excluded). The *t*-scores and *p*-values in the table were obtained from the T_Initial-EOT.

Abbreviation: AAL, automated anatomical labeling atlas; EOT, after 6 cycles for end of therapy assessment; Initial, baseline for staging; MNI, Montreal Neurological Institute;

Table S2. Peaks of the clusters of chemotherapy-induced changes in cerebral metabolism in DLBCL patients.

Brain Region (AAL2)	MNI coordinates			*t*	***p*-value$_{adj}$**	Cluster size
	x	y	z			
Gyrus rectus (R)	8	16	-24	-8.22	< 0.001	756
Lateral orbital gyrus (L)	-44	34	-16	-6.99	< 0.001	506

The x, y, z = coordinates according to the MNI stereotactic space for clusters showing the effects attributable mainly to chemotherapy, in the AAL2 atlas (cerebellum excluded). The *t*-scores and *p*-values in the table were obtained from the T_Initial-EOT.

Abbreviation: AAL, automated anatomical labeling atlas; EOT, after 6 cycles for end of therapy assessment; Initial, baseline for staging; MNI, Montreal Neurological Institute;

2. Detailed Description of the Comparison Process

In this section, chemotherapy will be referred to as *chemoTx*. Cancer and chemoTx status of the three *t*-statistics maps can be conceptually simplified as follows:

- T_HC-Initial: Cancer (+)
- T_HC-EOT: Cancer (±), ChemoTx (+)
- T_Initial-EOT: Cancer (-), ChemoTx (+)

In each *t*-statistics map, clusters showing significant increases or decreases in cerebral metabolism can be classified as follows:

Figure S1. Conceptual diagram illustrating cancer- and chemotherapy-induced changes in cerebral metabolism across the three t-statistic maps.

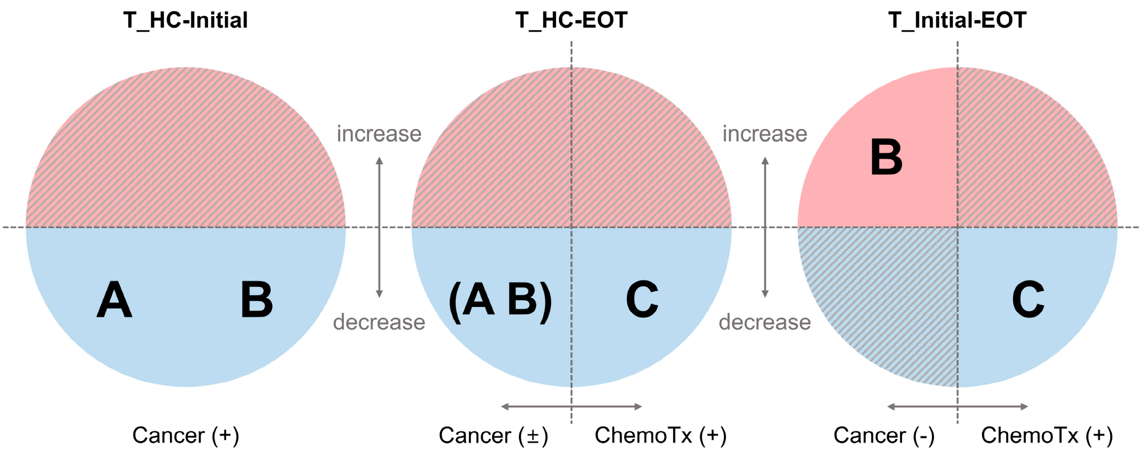

Semicircles separated by horizontal lines represent significant increases (red) and decreases (blue) of cerebral metabolism in each *t*-statistics map. Semicircles separated by vertical lines represent cancer-induced changes (left) and chemoTx-induced changes (right) in each *t*-statistics map. Three cluster types (A-C) can be observed: A, cancer-induced decrease (irreversible); B, cancer-induced decrease (reversible); C, chemoTx-induced decrease. Gray hatched areas represent regions that were either not observed or not considered.

Our objective was to distinguish cancer- and chemoTx-induced changes in cerebral metabolism observed in the T_Initial-EOT comparison. Overlap between T_Initial-EOT and T_HC-Initial was used

to identify cancer-induced changes. Overlap between T_Initial-EOT and T_HC-EOT was used to identify chemoTx-induced changes.

In the actual analysis. T_HC-Initial showed no significant increase, so decreases in T_Initial-EOT due to cancer resolution were not considered. T_HC-EOT also showed no significant increase, so increases in T_Initial-EOT due to chemoTx were not considered.

Thus, observable clusters in each *t*-statistics map were categorized as follows (brackets indicate effects presumed to be minimal):

- Decrease in T_HC-Initial: A, B
- Decrease in T_HC-EOT: (A, B), C
- Increase in T_Initial-EOT: B
- Decrease in T_Initial-EOT: C

Consequently, clusters showing an increase in T_Initial-EOT that overlapped with decrease in T_HC-Initial and exceeded an extent threshold of 200 voxels, were interpreted as reversible cancer-induced decreases in cerebral metabolism (Cluster B). Similarly, clusters showing a decrease in T_Initial-EOT that overlapped with decrease in T_HC-EOT and exceeded the extent threshold of 200 voxels, were interpreted as chemoTx-induced decreases in cerebral metabolism (Cluster C).
